# Understanding the role of religious beliefs in adherence to antiretroviral therapy among Pentecostal Christians living with HIV in sub-Saharan Africa: a scoping review

**DOI:** 10.1186/s12889-023-16616-5

**Published:** 2023-09-11

**Authors:** Ivo Nchendia Azia, Anam Nyembezi, Shernaaz Carelse, Ferdinand C. Mukumbang

**Affiliations:** 1 School of Public Health, University of the Western Cape, Robert Sobukwe Road Private Bag X17, Cape Town, 7535 Bellville South Africa; 2 Department of Social Works, University of the Western Cape, Cape Town, South Africa; 3 Department of Global Health, University of Washington, Seattle, USA

**Keywords:** People living with HIV, ART adherence, Antiretroviral therapy, Pentecostal Christians, Religious beliefs, Scoping review, Psychosocial factors, Sub-Saharan Africa

## Abstract

**Background:**

Optimum adherence to antiretroviral therapy (ART) is crucial in managing HIV. However, some people’s religious beliefs can influence how they deal with HIV and the psychosocial factors influencing their adherence to ART, such as disclosure, acceptance of HIV status, belief in ART, and depression. In sub-Saharan Africa (SSA), the role of religious beliefs in ART adherence is underexplored. We aimed to identify and conceptualize the literature on religious beliefs concerning ART adherence among Pentecostal Christians living with HIV in SSA.

**Methods:**

We conducted a scoping review of the literature on religious beliefs and ART adherence. We searched papers from PubMed, Web of Science, Medline, Sabinet, Academic Search Complete, CINAHL Plus, Health Source/Nursing Academic, Scopus, and Google Scholar and published papers from conference proceedings and dissertations. Data were extracted according to a predetermined population, concept, context framework, and eligibility criteria for selecting or rejecting studies. We used a narrative synthesis to summarize the data on evidence and the impact of religious beliefs on ART adherence.

**Results:**

Seven papers published between January 2010 and February 2022 met the inclusion criteria. Nineteen aspects of religious beliefs were identified as negatively influencing ART adherence, while eight aspects facilitated optimal adherence. “Being saved” or “born again” enhanced coping strategies for optimal adherence through actions such as less alcohol use, fidelity to a sexual partner(s), disclosure, acceptance of HIV status, reduced depression, and facilitated PLHIV to access social support from church members or other institutions.

**Conclusion:**

Religious beliefs are integral to Pentecostal Christians living with HIV and affect their adherence to ART. While some Pentecostal Christians living with HIV on ART use their religious beliefs and practices to access psychosocial support from other church members or organizations and achieve good clinical outcomes, others apply their religious beliefs and practices differently and compromise their commitments to taking ART as prescribed, thus experiencing poor viral suppression and clinical outcomes. However, more research is required to understand and theorize how religious beliefs impact ART adherence among Pentecostals living with HIV to inform guidelines for practitioners.

**Supplementary Information:**

The online version contains supplementary material available at 10.1186/s12889-023-16616-5.

## Background

Sub-Saharan Africa (SSA) continues to endure a disproportionate burden of HIV/AIDS, with an estimated 25.3 million people living with HIV (PLHIV) [1]. Many countries in the region are thus implementing the UNAIDS targets to end AIDS by 2030 [2]. The UNAIDS's 95–95-95 global targets recommend that by 2030 all countries should have 95% of PLHIV know their HIV status, 95% of those diagnosed with HIV enroll in ART, and 95% of those on ART achieve viral suppression [3].

Presently, more than 17.9 million PLHIV are known to be on ART in SSA. This outcome was partly achieved through the extensive adoption of the 2014 UNAIDS’s 90–90-90 targets and the Universal Test and Treat guidelines, which emphasize that every person who tests positive for HIV be initiated on ART irrespective of their CD4 count or WHO clinical staging [4]. However, in SSA, many PLHIV are still not on ART. In West and Central Africa particularly, out of 2.6 million PLHIV on ART, just about 39% of them have had suppressed viral loads because of non-adherence to ARTs [5].

Remarkable health benefits are being derived by PLHIV, public health, and healthcare providers from the use of ART in the control and management of HIV [6–8]. Clinical markers such as viral loads and CD4 lymphocyte counts are usually used to assess the clinical efficacy of ART in PLHIV [9]. PLHIV who optimally adhere to taking at least 95% of ART as prescribed are known to experience undetectable viral loads and high CD4 counts [6, 8, 10]. Optimal adherence to ART is also known to prevent drug resistance and the need to switch patients to alternative treatment options that are more costly and difficult to deliver in healthcare settings in developing countries [11–13]. Nevertheless, the benefits of good adherence to ART hinge on PLHIV’s ability to submit to a lifetime obligation of taking at least 95% of ART as prescribed [12, 14, 15]. Therefore, it is paramount that the factors that impact patients' adherence to ART are well understood and considered to achieve sustained treatment outcomes.

Practically, optimal adherence to ART is a challenging and ongoing process that depends on many factors, such as socio-economic, cultural, health systems, and therapy-related factors [16–18]. Commonly reported patient-related factors impacting adherence to ART are substance abuse, emotional distress, treatment fatigue, and religious beliefs [19–22]. Religion is essential in many people's lives, as over 88.7% of the world’s population professes at least one kind of religion [23]. In Africa, 47% of Africans are evangelized, and 49% are Christianized [24], and of the known Christian denominations, Pentecostal Christians are the fastest and the largest growing denomination [25]. A peculiar attribute of the Pentecostal Christians is their religious belief and practice of using the power of the “Holy Spirit” to heal people, especially those suffering from chronic diseases like HIV [26–28]. Nonetheless, little is known about other religious beliefs pertinent to adherence to ART among Pentecostal Christians on HIV treatment.

Psycho-social factors have often been used to explain the adherence behaviors of people on chronic medications such as ART [29–31]. Some people's religious beliefs have been reported to affect how they deal with HIV and other psycho-social factors influencing their treatment adherence, such as believing in the ART, disclosing, and accepting their HIV status, HIV stigma and discrimination, depression, and anxiety [32–34]. Some studies assessing the impact of religion on ART adherence have found a positive correlation between religious beliefs and practices such as praying, attending regular prayer meetings, and church services [33, 35, 36]. Other studies have also demonstrated that religious beliefs have enabled Christians living with HIV to cope with common mental health problems such as depression, thus enhancing their ART adherence [34, 37, 38]. Religious beliefs have also been reported to help Christians living with HIV to access social support from church members and other institutions to enhance their adherence to HIV treatment [35, 37, 39].

The impact of religious beliefs on the prevention, management, and care of PLHIV in sub-Saharan Africa has also been acknowledged from the onset of the HIV pandemic [40]. Faith-linked organizations such as Islamic Relief, Tear Fund, Caritas Internationalist, World Conference of Religion for Peace, and the International Network of Religious Leaders living with HIV, are now partnering and assisting major stakeholders like the WHO and UNAIDS with resources to curb HIV transmission and strengthen adherence to ART and support to PLHIV [40].

On the contrary, the activities of some faith-based organizations have been negatively associated with information about HIV and adherence to ART among Christians living with HIV. Aspects of religious beliefs, like receiving healing through fasting and prayers, believing in the healing powers of pastors and prophets, or believing that HIV is spiritual and the demons causing it can be cast out through religious rituals, have been reported as barriers to adherence to ART [17, 28, 33]. The belief of some prominent spiritual leaders in using unconventional healing methods like drinking petrol, eating grass and snakes, and spraying insecticides on their church members to heal them from various health problems has been reported [41]. Healthcare workers have also reported the belief in the potency of spiritual healing over orthodox ART in different settings [42–44]. The use of unusual religious beliefs and rituals for the treatment of HIV have equally been well documented as barriers to adherence to ART [27, 28, 45], thus thwarting the medical community’s efforts to enroll and maintain Christians living with HIV on HIV treatment [35, 36, 46].

To mitigate religion-related barriers to adherence to ART among Christians living with HIV, knowledge of religious beliefs and practices hindering optimal adherence has often been employed in designing ART adherence interventions, especially in the counseling components of such interventions. It has also been recommended that healthcare professionals discuss spirituality or religious issues with individuals and establish their role in managing patients [34].

The literature describing religious-embedded adherence interventions to improve ART adherence among Christians living with HIV is generally limited [36, 47]. Previously reported data on the negative impact of religious beliefs on ART adherence outcomes, especially in SSA, have been predominantly anecdotal accounts obtained from studies with limited respondents and not based on established empirical methods [33]. There have also been calls from some stakeholders to embed religion into HIV-care to improve the physical and mental outcomes of PLHIV. No﻿netheless, the varied nature of the support from various stakeholders conducting spiritual assessments and providing spiritual care in healthcare settings have been reported as setbacks in such interventions [47]. Alternatively, more collaboration with alternative medicine providers such as Pentecostal Christian religious leaders, spiritual prophets, and pastors has also been recommended as a way forward to fortifying adherence to ART among Christians living with HIV [26, 28, 48].

Worldwide, many people belong to organized religions, and depending on their respective religious affiliations, their religious beliefs and practices can influence their healthcare beliefs and practices [49]. While some people on ART have used their religious beliefs and practices to access psychosocial support from other church members or organizations and achieved good clinical outcomes, others have applied theirs differently, thus compromising their commitments to taking ART as prescribed, leading to poor clinical outcomes [33]. Relevant information on the religious beliefs and practices of Christians living with HIV can be integrated into their HIV treatment plans. Such information can also be helpful to healthcare professionals in developing better ART adherence strategies and consequently obtaining optimum health outcomes for PLHIV. Nonetheless, there is still a paucity of evidence, particularly in SSA, concerning religious beliefs relevant to ART adherence among Christians living with HIV [37]. Consequently, we employed a scoping review methodology to explore and hypothesize the relationships between religious beliefs and adherence to ART among Pentecostal Christians living with HIV in SSA.

## Methods

The authors first developed a scoping review protocol [50] as part of a larger study to understand the adherence behaviors of Pentecostal Christians living with HIV to ART to enhance guidelines development [51]. However, we did not register the protocol before writing this scoping review. According to Colquhoun et al. [52], a scoping review is a form of knowledge synthesis that addresses an exploratory research question aimed at mapping key concepts, types of evidence, and gaps in research related to a defined area or field by systematically searching, selecting, and synthesizing existing knowledge. This scoping review was conducted following the methodological steps suggested by Arksey and O’Malley [53]: (i) identification of the research question; (ii) identification of relevant studies for the scoping review; (iii) selection of the literature for the scoping review; (iv) charting the data; and (v) collating, summarizing and reporting the results. The PRISMA Extension for Scoping Reviews (PRISMA-ScR): Checklist and Explanation from Tricco et al. [54] was used to ensure that the scoping review was reported accordingly (see the Supplementary File 1). We used a narrative synthesis to summarize the data extracted to report on the nature of existing evidence and the impact of religious beliefs on ART adherence among Pentecostal Christians living with HIV in SSA.

### Research question

In the first years of ART delivery, access to ART was unequally distributed in SSA, and as few as 7 000 individuals in SSA were receiving ART in the early 2000s [55]. At the same time, the international community did not support the scale-up, as treating HIV on a large scale was still untested [56]. However, by 2005 it was evident that treating HIV on a large scale was highly attainable hence the international community committed to achieving universal access to ART by 2010 [56]. Since then, some of the significant challenges many countries face in SSA in treating HIV have been expanding access and sustaining optimal adherence to ART. Presently, many PLHIV are still not on ART in West and Central Africa, and out of about 2.6 million PLHIV on ART, just about 39% of them have had suppressed viral loads due to poor adherence to ART [5]. Evidence shows that optimal levels of adherence to ART are not frequently attained among PLHIV due to many factors, including religious beliefs [22, 28, 33].

Generally, some people's religious beliefs can influence how they deal with HIV and the psychosocial factors influencing their adherence to ART, such as believing in ART, disclosing, and accepting HIV status, stigma and discrimination, depression, and anxiety [32–34]. Also, in SSA, among other denominations, Pentecostal Christians are well known for their strong belief in using the power of the Holy Spirit to heal diseases such as HIV [26–28]. However, within the past decade of expanding access and sustaining adherence to ART in SSA, little is known about the role of religious beliefs among Pentecostal Christians living with HIV.

Hence, this scoping review answered the following research question: “How do religious beliefs impact adherence to ART among Pentecostal Christians living with HIV in SSA”? The study explored the following sub-questions: (1) What is the impact of religious beliefs on ART adherence among Pentecostal Christians living with HIV in SSA?” (2). How do religious beliefs relate to the psycho-social factors that impact adherence to ART among Pentecostal Christians living with HIV in SSA?

### Identification of relevant studies

Relevant studies for the scoping review were identified through a systematic search of nine databases using Boolean phrases developed and tested using PubMed when writing the protocol for this scoping review [50]. We searched the literature from the following databases: Web of Science, EMBASE, PubMed/Medline, Psych-ARTICLES, Academic Search Complete, Cumulative Index of Nursing, Allied Health (CINAHL), EBSCOhost interface, and Scopus. A literature search was done using free-text words on bibliographical search engines such as the WHO repository, National Health Departments, Academia.edu, and Google Scholar. Gray literature was equally searched from Theses and Dissertations. Open Access and published reports from conference proceedings were searched equally. The reference list of included studies was also searched to obtain relevant literature.

The search process was iterative and has been included in the study's results section. The ‘MeSH term,’ abstract or title table, keyword, and text word tabs were selected when searching for studies and articles. In the process of searching, search terms were combined using the Boolean technique (AND, OR) as follows: (adherence) AND (faith) OR (Pentecostal*) OR (Christian*) OR (religious* beliefs) OR (Psycho-social factors) AND (antiretroviral treatment) OR (antiretroviral medication) OR (HAART) OR (HIV medication) OR (antiretroviral therapy).

### Pilot searches

We conducted a pilot search with an expert at the University of the Western Cape in PubMed to identify potential problems that had to be addressed before conducting a final search of relevant studies and articles. A record of all the searches was kept and updated during the search process to monitor when the same search terms were used in other databases. Eligible studies and articles were uploaded into the Zotero software, and any duplicates found in the selected studies were removed.

### Study selection

To ensure that the findings of this review are based on recent and relevant evidence, we selected studies published between January 2010 and February 2022, given that research is constantly growing, and new studies are being published regularly. Only studies that met the inclusion criteria were selected, as presented in Table 1.

**Table 1:** The PCC framework and eligibility criteria for selecting or rejecting studies

Criteria	Determinants	Inclusion criteria	Exclusion criteria
Population	Adult Pentecostal Christians living with HIV	Articles and studies reporting on adult Pentecostal Christians living with HIV who are 18 years old and above	Articles and studies reporting on other religions or not reporting on adult Christians living with HIV
Content	Barriers or facilitators of Adherence to ART	Articles or studies reporting on Christian religious beliefs as barriers or facilitators of ART adherence and how religious beliefs relate to the psycho-social factors that impact adherence to ART	Articles or studies not reporting on Christian religious beliefs as barriers or facilitators of ART adherence and how religious beliefs relate to the psycho-social factors that impact adherence to ART
Context	sub-Saharan Africa	Articles and studies reporting on Christian religious beliefs as barriers or facilitators of ART adherence in sub-Saharan Africa	Studies and articles report on Christian religious beliefs as barriers or facilitators of ART adherence in other parts of the world
Sources of evidence	Empirical and gray literature	Evidence from empirical and gray literature, such as government documents, NGO reports, and academic dissertations from all study designs, that meet the selection criteria	Evidence literature reviews, study protocols, editorials, commentaries, and news reports will not be selected
Others	Language	All articles and studies which meet the selection criteria and are written in English	All articles and studies which meet the selection criteria but are written in other languages
	Time	All articles and studies which meet the selection criteria and were published between 2010 and February 2022	All articles and studies which were published before 2010 and after February 2022

**Fig. 1:**
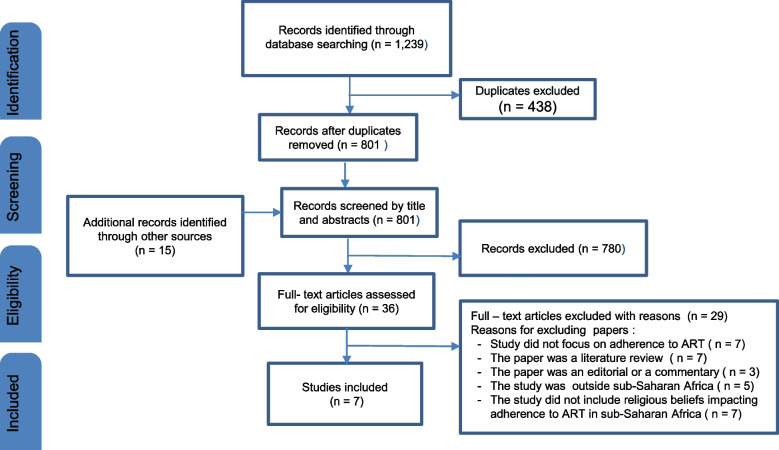
The PRISMA flow diagram illustrates the study selection process

The study selection process is shown in the PRISMA flow diagram in Fig. 1. Two reviewers screened eligible studies at two levels. The first part of the screening entailed a double and independent screening of the titles and abstracts of studies depending on whether the studies initially met the inclusion criteria described in Table 1. During the screening process, the authors of papers that met the inclusion criteria, but full-text copies of the paper were not available online were contacted for the full-text copies. However, for unsuccessful requests, we excluded the articles from this study. A screening guide was developed from the PCC framework and eligibility criteria for selecting or rejecting studies (Table 1) and provided instructions to help screeners screen objectively. The screening of the studies was piloted on the screening of 11 abstracts of articles and a full-text screening of five articles.

### Charting the data

We used an Excel form to capture data from studies that met the inclusion criteria. Two reviewers independently charted the data into the data capturing form under the following headings: the name of authors and date of publication, the title of the study, the aim of the study, setting of the study, the study population, sampling method used, the study design, data collection methods, study findings, and the conclusion drawn by the study. A preliminary test of the appropriateness of the data charting form was done using three randomly picked studies, and the feedback obtained from the preliminary test conducted on the data charting form was used to improve the quality and accuracy of the data capturing tool.

### Collating, summarizing, and reporting the results

We grouped and summarized studies focusing on religious beliefs impacting adherence to ART among Pentecostal Christians living with HIV in SSA first. We also considered studies on religious-embedded ART adherence interventions carried out in SSA and their impacts on adherence to ART among Pentecostal Christians living with HIV. Finally, we grouped and summarized studies reporting on how religious beliefs related to the psychosocial factors impacting adherence to ART among the Pentecostal Christians living with HIV in SSA. We then described the results of the scoping review in line with the research questions and the purpose of the study.

**Fig. 2:**
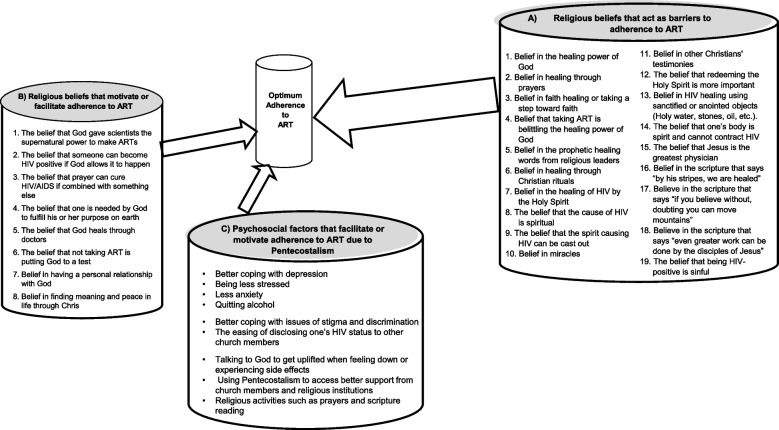
Study findings

Using the factors and the relationships between aspects of religious beliefs and identified psychological barriers to ART adherence, we constructed a comprehensive model to hypothesize a multidimensional and dynamic relationship (Fig. 2). Our construction of this model was based on creative abduction, whereby new knowledge is generated to explain observed events [57]. Creative abduction unifies a given body of knowledge with multiple correlated outcomes toward theory formation [57].

## Results

We obtained 1,239 hits from 8 databases searched (see Table 2). Of this number, 438 were duplicates and were removed, while 801 papers were retained. After screening for possible relevant titles and abstracts of the retained 801 papers, 780 did not qualify for full-text screening and were removed because either their titles or abstracts were irrelevant to the study. However, 21 papers qualified for full-text screening and were retained. A further search was conducted on Google Scholar, which yielded nine other papers since one paper was excluded because we could not retrieve its full text. Later, a reference list search was conducted on the papers that qualified for full-text screening, and six additional studies were found and added to the 21 initial papers eligible for the full-text screening. Thus, 36 papers (21 + 9 + 6 = 36) passed through full-text screening and 7 of them met the inclusion criteria and were included in the review. Figure 1 provides a flow chart of the study selection process.

**Table 2:** Databases searched, and the number of articles obtained

Database Searched	Number of Articles Obtained
PubMed	170
Web of Science	282
Medline	118
Sabinet	405
Academic Search complete	142
CINAHL Plus	66
Health Source: Nursing/Academic	40
Scopus	16
**Total**	**1, 239**

### Characteristics of the studies included in the review

All articles retained reported on studies that were published between 2010 and 2021. Of the studies, one was a dissertation, and six were published in peer-reviewed journal articles. The distribution of the papers by year of publication shows that three of the studies were published between 2010 and 2015, while four were published between 2015 and 2021. Two of the studies [28, 58] were conducted in Zimbabwe, one study [59] was conducted in South Africa, followed by two studies [35, 37] conducted in Nigeria, and two others [33, 60] were conducted in Uganda. Four studies used quantitative designs, while three studies used qualitative designs. A summary of relevant information extracted from the seven eligible studies in connection to religious beliefs impacting ART adherence among Pentecostal Christians living with HIV in SSA is provided in Supplementary file 2.

### Study findings

We classified the religious beliefs impacting adherence to ART among the Pentecostal Christians living with HIV in SSA under three headings, (A) Religious beliefs as barriers to adherence to ART; (B) religious beliefs as facilitators or motivators to ART adherence, and (C) Psychosocial factors that facilitate or motivate adherence to ART due to Pentecostalism. Figure 2 shows the study findings.

#### Religious beliefs that act as barriers to adherence to ART

Three studies reported nineteen aspects of religious beliefs as barriers to ART adherence (see Fig. 2). The belief in the healing power of God was the most reported barrier to adherence to ART in the group [28, 33, 58]. Two studies reported four aspects of religious beliefs as barriers to ART: the belief in obtaining healing through prayers, the belief in faith healing or taking a step of faith, the belief that taking ART as belittling the healing power of God, and the belief in the prophetic healing words from religious leaders [28, 33]. The other aspects of religious beliefs reported as barriers to adherence to ART are reported in Fig. 2.

#### Religious beliefs that facilitate or motivate adherence to ART

Eight religious beliefs were reported as motivating or facilitating Pentecostal Christians living with HIV to take ART as prescribed in three studies [33, 35, 58]. The most frequently reported religious belief as a facilitator of ART adherence was the belief that God gave scientists the supernatural power to make ART, which was reported in two studies [33, 58]. The other factors found under this category are reported in Fig. 2.

#### Psychosocial factors that facilitate or motivate adherence to ART due to Pentecostalism

Religious beliefs were associated with three psychosocial factors impacting adherence to ART among Pentecostal Christians living with HIV in SSA; mental health symptoms, social coping, and religious coping, as illustrated in Fig. 2. Three studies reported three improved mental health symptoms linked to Pentecostalism, which are better coping with depression, anxiety, and stress [33, 37, 59]. One study reported two psychosocial coping aspects of adherence to ART among the Pentecostal Christians living with HIV that improved coping with stigma and discrimination and the side effects of ART. The same study also reported one psychosocial factor of adherence to ART to be the ease of disclosing one’s HIV status to other church members [33]. Talking to God to get uplifted when feeling low was reported as a psychosocial coping strategy in the study by Tumwine et al. [33]. In the study by Ayuk et al. [37], religious coping aspects that were reported to have facilitated adherence to ART among Pentecostal Christians living with HIV were: receiving good support and teachings from church members and religious institutions, having a positive psychological impact on private religious activities such as prayers and scripture reading, and a reduction in the use of negative religious coping strategies such as feeling abandoned by God or members of a religious community. In the same study, looking for a stronger connection with God or seeking God’s love and care as well as focusing on religion to stop worrying about other problems were also reported as religious coping aspects that facilitated good adherence to ART. The psychosocial factors that facilitated or motivated adherence to ART due to Pentecostalism are shown in Fig. 2.

**Fig. 3:**
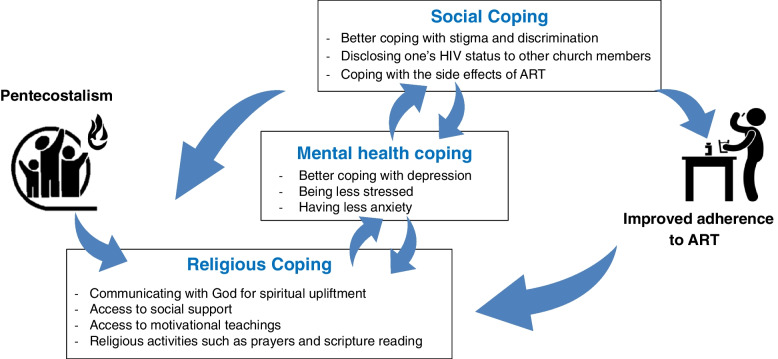
The hypothesized relationship between religious beliefs and the psychosocial factors influencing adherence to ART among Pentecostal Christians living with HIV in sub-Saharan Africa

The hypothesized relationship (model) of the dynamic and multidirectional influence of Pentecostalism on ART adherence for some Pentecostal Christians living with HIV in sub-Saharan Africa is shown in Fig. 3.

## Discussion

This scoping review was designed to provide insight into the relevance of religious beliefs among Pentecostal Christians living with HIV in SSA concerning ART adherence. Evidence shows a multifaceted impact of religious beliefs on ART adherence among Pentecostal Christians living with HIV. In the first facet, religious beliefs such as religious rituals, prayers, testimonies, prophecies, biblical scriptures, and the influence of religious leaders promoting spiritual healing and deliverance over conventional ART hinders adherence to ART among Pentecostal Christians living with HIV. This finding draws attention to the potential negative role that religious beliefs can play in adherence to ART among some Pentecostal Christians living with HIV. Previous studies [19, 22, 42] reported the detrimental potential of religion to ART adherence among some highly religious people based on their unweaving belief in healing through prayers, holy water, and other faith-based healing rituals. In this review, Tumwine et al. [33] also identified teachings and prophecies from religious leaders and supporting Biblical scriptures to have led Pentecostal Christians living with HIV to feel that God and their faith in Him, and not ART, would heal them.

One of the current global targets of the UNAIDS in ending HIV by 2030 requires that PLHIV on ART achieve a 95% suppressed viral load [3]. Given this objective, knowledge concerning what motivates or facilitates optimum adherence to ART or developing strategies to strengthen adherence to ART is relevant. Four aspects of religious beliefs identified to facilitate good adherence to ART that could be harnessed to improve ART adherence among people with Pentecostal beliefs living with HIV are: (1) God needs everyone to fulfill his or her purpose on earth, (2) God heals through doctors, (3) not taking ART is putting God to the test, and (4) God gave scientist the knowledge to manufacture ART. This finding shows that religious beliefs are not always unhelpful or act as deterrents in sustaining long-term adherence to ART among Pentecostal Christians living with HIV. To this end, we suggest that spirituality should be considered when designing adherence interventions, especially in the counseling components of such interventions. It has also been recommended that clinicians discuss spirituality or religion with individual patients and determine its role in managing HIV treatment in patients case-by-case [34].

Our finding shows that religious beliefs also influence the psychosocial factors motivating or facilitating good adherence to ART among Pentecostal Christians living with HIV. We identified that positive coping with depression, anxiety, and stress led to improved adherence to ART among some Pentecostal Christians living with HIV. However, positive coping with problems of medication side effects of ART was also reported in the study by Ayuk et al. [37], while positive coping with stigma and discrimination, which are the most frequently reported reasons why patients default on ART, were also reported in Tumwine et al. [33]*.* In the same study, the conventional belief in God as a source of comfort and support was reported to enable Pentecostal Christians living with HIV to cope with mental health symptoms associated with inadequate adherence as opposed to fundamentalist beliefs in the healing power of God which causes some Pentecostal Christians living with HIV to abandon ART. Pentecostalism also enabled some Pentecostal Christians living with HIV to access social support from church members and other institutions, thus enhancing adherence to ART [35, 37, 39]. Koenig [61] found a positive association between positive religious coping and adherence to ART and suggested that positive coping may be linked to improved attendance to scheduled medical appointments and better compliance with ART. Conversely, negative coping, like feeling abandoned by God, reported among adolescents to be associated with poor adherence to ART by Lyon et al. [62] was not reported in any study in the review. This phenomenon could be explained by the fact that most Pentecostal Christians have an unweaving belief that God can never forsake or abandon them, no matter their situation.

Knitting the relationship between Pentecostalism, ART-related psychosocial issues, and ART adherence among Pentecostal Christians living with HIV through abduction provides a comprehensive picture (Fig. 3). This hypothesized relationship (model) shows Pentecostalism's dynamic and multidirectional influence on ART adherence for some Pentecostal Christians living with HIV. The model demonstrates the relationship between Pentecostalism and ART adherence being mediated by Pentecostalism, addressing psychosocial issues related to ART adherence and improving patients’ resilience to social adversity such as stigma and discrimination. In turn, the achieved resilience and self-efficacy reinforce patients' beliefs (based on Pentecostalism and its practices), fortifying their resilience and self-efficacy. Such a model can be helpful in further exploring and refining these relationships and designing robust interventions to address ART adherence among this population. The need for such a model is critical because of the conspicuous dearth of religious-embedded ART adherence studies in SSA, particularly among Pentecostal Christians.

### Strengths and limitations of this study

The strength of this scoping review emanates from the study’s ability to provide a practical way to identify, explore and map the literature on religious beliefs and how they impact adherence to ART among Pentecostal Christians living with HIV in SSA. This strength is compounded through the adductive elicitation of a tentative theory based on information from the identified literature. Another point of strength comes from the broad search of data from eight databases and the search of grey literature on Google Scholar, Theses and Dissertations libraries, Open Access, and published reports from conference proceedings. Furthermore, the blinded process used in the screening of the articles minimized bias and increased the credibility of the screening process.

A significant weakness of the scoping is that only English articles were used in the study due to limited resources for translation. This restriction may have increased the possibility of missing out on studies reporting religious beliefs impacting adherence to ART among Pentecostal Christians living with HIV that have been published in other languages used in SSA.

## Conclusion

Religious beliefs have a multifaceted impact on adherence to ART among some Pentecostal Christians living with HIV in SSA. More religious beliefs negatively influence adherence to ART than those that motivate or facilitate good adherence in this scoping review. While some Pentecostal Christians living with HIV on ART use their religious beliefs and practices to access psychosocial support from other church members or organizations and achieve good clinical outcomes, others apply their religious beliefs and practices differently and compromise their commitments to taking ART as prescribed, thus experiencing poor viral suppression and clinical outcomes.

However, there is still a premature understanding of the nature of religious beliefs and how they impact ART adherence among people with Pentecostal beliefs living with HIV in SSA. We elicited a model to inform further thinking on the role(s) of a system of beliefs, moral values, or codes of conduct and practices and how and why they influence the decisions of some Pentecostal Christians living with HIV regarding testing, acceptance of HIV test results, initiating ART and adhering to the treatment. A refined version of such a model can inform interventions and ways of improving ART adherence among Pentecostal Christians living with HIV and other PLHIV who share their religious beliefs.

### Supplementary Information

**Additional file 1.** Preferred Reporting Items for Systematic reviews and Meta-Analyses extension for Scoping Reviews (PRISMA-ScR) Checklist.

**Additional file 2.** Relevant data was extracted from the seven eligible studies on religious beliefs impacting adherence to ART among Pentecostal Christians in sub-Saharan Africa.

## Data Availability

This published article and its supplementary information files include all data generated or analyzed during this study.

## Abbreviations

ART: Antiretroviral therapy
SSA: Sub-Saharan Africa
HIV: Human immunodeficiency virus
AIDS: Acquired immunodeficiency syndrome
UNAIDS: Joint United Nations Program on HIV/AIDS
CD4: Cluster of differentiation
WHO: World Health Organization
PLHIV: People living with HIV.
PRISMA-ScR: Preferred Reporting Items for Systematic reviews and Meta-Analyses extension for Scoping Reviews
JBI: Joanna Briggs Institute
PCC: Population, concept, and context
MeSH: Medical Subject Headings
HAART: Highly active antiretroviral therapy
NGO: Non-governmental organization
