# Supplementary material for: Understanding the role of religious beliefs in adherence to antiretroviral therapy among Pentecostal Christians living with HIV in sub-Saharan Africa: a scoping review
Source: BMC Public Health. 2023 Sep 11;23:1768. doi: 10.1186/s12889-023-16616-5 (PMC10494378; doi:10.1186/s12889-023-16616-5)
Supplement: Supplementary file 2 — Additional file 2. Relevant data was extracted from the seven eligible studies on religious beliefs impacting adherence to ART among Pentecostal Christians in sub-Saharan Africa. [file 12889_2023_16616_MOESM2_ESM.docx]

| **Author and date** | **Title of study** | **Study setting** | **Study design** | **Study participants** | **Study sample size** | **Religious beliefs reported as**  **barriers to adherence**  **to ART** | **Religious beliefs were reported as Motivators of ART adherence** | **Reported relationships between religious beliefs and psychosocial factors impacting adherence to ART** |
| --- | --- | --- | --- | --- | --- | --- | --- | --- |
| Amadi et al. 2020 | Religion and adherence to antiretroviral medication: is there a link? | University of Nigeria Teaching Hospital (UNTH) | Cross-sectional study | Adults females and males living with HIV of 32 to 42 years old | 140 | Not reported | - Belief in having a personal relationship with God  - Finding meaning and peace in life through belief | Not reported |
| Ayuk et al. 2017 | Influence of Spirituality and Religion on Adherence to Highly  Active Antiretroviral Therapy in Adult HIV/AIDS  Patients in Calabar, Nigeria | University of Calabar Teaching Hospital, Calabar Nigeria | Cross-sectional descriptive study | Adults females and males living with HIV of 20 to 69 years old | 370 | Not reported | Not reported | **Belief of being a born again or being saved influenced the engagement in good adherence behaviors as:**   - lowering alcohol use   **Being born again positively influenced coping aspects of adherence to ART** as:   - Better coping with depression symptoms and mental health problems - Coping with problems of side effects of medication - -Being less stressed and worried other problems - Better coping with issues of stigma and discrimination - Belief of being born again or being saved influenced religious coping aspects of adherence to ART as: - Having better support and teachings from religious institutions - Having a positive psychological impact of private religious activities such as prayers and scripture reading |
| Kisenyi et al. 2011 | Religiosity and Adherence to Antiretroviral Therapy Among Patients Attending a Public Hospital-Based HIV/AIDS Clinic in Uganda | Mulago Hospital in Kampala, Uganda | Descriptive quanitative | 220 males and females living with HIV of 15–45 years old | 220 | Not reported | Not reported | **Being born again positively influenced coping aspects of adherence to ART** as   - Being less stressed and worried other problems |

| Mutambara et al. 2021 | Harmonizing religion and health: an exploration of religious reasons for defaulting ARVs among people living with HIV and AIDS in Gweru, Zimbabwe | Gweru, Zimbabwe | Qualitative survey | 15 Adult males and females living with HIV,  of 17 to 53 years old, 6 adherence counselors, 29 to 57 years old and 4 of religious leaders 34 to 70 years old | 25 | - Believing in the healing power of God - Believing in healing through prayer - Believing in healing through Christian rituals - Believing in faith healing or taking a step of faith Believing in the healing of HIV by the Holy spirit - Believing in HIV healing through the use of sanctified or anointed objects (Holy water, stones, oil) - Believing that the cause of HIV is spiritual - Believing that the spirit causing HIV can cast out- Believing that taking ART is belittling the healing power of God | Not reported | Not reported |
| --- | --- | --- | --- | --- | --- | --- | --- | --- |
| Peltzer et al. 2011 | Spirituality and Religion in Antiretroviral Therapy (ART) in Kwazulu-natal, South Africa: A Longitudinal Study | Kwazulu-natal, South Africa | Longitudinal design | 735 Adult males and females living with HIV, 18 to 67 years old | 735 | - Not reported | Not reported | **Belief of being a born again or being saved influenced the engagement in good adherence behaviors as:**   - lowering alcohol use   **Being born again positively influenced coping aspects of adherence to ART** as:   - Better coping with depression symptoms and mental health problems |
| Tumwine et al. 2012 | Reasons Why High Religiosity Can Co-exist with and Precipitate Discontinuation of Anti-retroviral Therapy among Different HIV Clients in Uganda: An Exploratory Study | Kampala, Uganda | Qualitative survey | 39 Adult males and females living with HIV 20 to 69 years old | 39 | - Believing in the healing power of God - Believing in healing through prayer - Believing in faith healing or taking a step of faith - Believing in a miracles - Believing in religious testimonies - Believing that redeeming the Holy spirit is more important - Believing that taking ART is belittling the healing power of God - Believing that one’s body is spirit and cannot contract HIV - Believing in prophetic healing words from Religious leaders - Believing that Jesus is the greatest Physician - Believing in the scriptures that says by his stripes we are healed - Believing in the scriptures that if you belief without doubting you can move mountains - Believing that even greater work can be done by the disciples of Jesus. - Believing that being HIV positive results from Satan. | - Believing that God gave scientists the supernatural power to make ARVs , - Believing that prayer can cure HIV/AIDS if combined with something else , - Believing one is needed by God to fulfill his or her purpose on earth - Believing that God heals through doctors - Believing that not taking your ART is putting God to a test | **Belief of being a born again or being saved influenced the engagement in good adherence behaviors as:**   - lowering alcohol use - Limiting the number of sexual partners - abstinence from sex - being faithful to partners in relationships - curbing womanizing - lowering smoking   **Being born again positively influenced coping aspects of adherence to ART** as:   - Better coping with depression symptoms and mental health problems - Taking to God to get lifted when feeling low - Being less worried about death and financial problems - Easing disclosure of HIV status to other church members |
| Moyo, 2018 | An assessment of the influence of Christian faith on art adherence for Christians living with HIV at ZNNP+ Gweru urban | ZNNP+ Gweru Urban, Zimbabwe | Qualitative | 13 Adults  males and females living with HIV, 18 to 55 years old | 13 | - Believing in the healing power of God | - Believing that God gave scientists the supernatural power to make ARVs - Believing that one can become HIV positive if God allows it | Not reported |
